# A quantitative framework reveals traditional laboratory growth is a highly accurate model of human oral infection

**DOI:** 10.1073/pnas.2116637119

**Published:** 2022-01-06

**Authors:** Gina R. Lewin, Kendall S. Stocke, Richard J. Lamont, Marvin Whiteley

**Affiliations:** ^a^School of Biological Sciences, Georgia Institute of Technology, Atlanta, GA 30332;; ^b^Emory–Children’s Cystic Fibrosis Center, Atlanta, GA 30322;; ^c^Center for Microbial Dynamics and Infection, Georgia Institute of Technology, Atlanta, GA 30332;; ^d^Department of Oral Immunology and Infectious Diseases, School of Dentistry, University of Louisville, Louisville, KY 40202

**Keywords:** *Porphyromonas gingivalis*, metatranscriptomics, periodontitis, laboratory models

## Abstract

Bacterial behavior and virulence during human infection is difficult to study and largely unknown, as our vast knowledge of infection microbiology is primarily derived from studies using in vitro and animal models. Here, we characterize the physiology of *Porphyromonas gingivalis*, a periodontal pathogen, in its native environment using 93 published metatranscriptomic datasets from periodontally healthy and diseased individuals. *P. gingivalis* transcripts were more abundant in samples from periodontally diseased patients but only above 0.1% relative abundance in one-third of diseased samples. During human infection, *P. gingivalis* highly expressed genes encoding virulence factors such as fimbriae and gingipains (proteases) and genes involved in growth and metabolism, indicating that *P. gingivalis* is actively growing during disease. A quantitative framework for assessing the accuracy of model systems showed that 96% of *P. gingivalis* genes were expressed similarly in periodontitis and in vitro midlogarithmic growth, while significantly fewer genes were expressed similarly in periodontitis and in vitro stationary phase cultures (72%) or in a murine abscess infection model (85%). This high conservation in gene expression between periodontitis and logarithmic laboratory growth is driven by overall low variance in *P. gingivalis* gene expression, relative to other pathogens including *Pseudomonas aeruginosa* and *Staphylococcus aureus*. Together, this study presents strong evidence for the use of simple test tube growth as the gold standard model for studying *P. gingivalis* biology, providing biological relevance for the thousands of laboratory experiments performed with logarithmic phase *P. gingivalis*. Furthermore, this work highlights the need to quantitatively assess the accuracy of model systems.

## Significance

Most microbial knowledge comes from experiments in laboratory models, despite the assumption that these artificial systems alter microbial physiology relative the native environment. We tested this assumption for an oral pathogen, *Porphyromonas gingivalis*, using 93 metatranscriptomes from periodontally healthy and diseased patients and 122 transcriptomes from experimental models. We discovered that a simple in vitro model, midlogarithmic growth in rich media, highly recapitulates *P. gingivalis* gene expression in the human oral cavity, outperforming other models, including a murine infection model. These results support the biological relevance of decades of laboratory experiments with this pathogen and validate an accessible experimental model for studying *P. gingivalis* biology. Furthermore, these data provide a conceptual framework for understanding in situ gene expression across microbes.

The ultimate goal of the vast majority of microbiology research is to understand the processes that shape microbial behavior, ecology, and evolution, ranging from studies of the microbial role in pathogenesis to the microbial contribution to nutrient cycling in the oceans. Experimental laboratory models such as in vitro culture, microcosms, and animal models are the workhorses of these studies and have greatly advanced our understanding of microbial physiology. However, the relationship between an experimental model system and a microbe’s native environment is often not well understood, and recent work has shown that the in situ gene expression signature is distinct from that in experimental model systems for some opportunistic pathogens (1–3).

Microbes in the oral cavity involved in periodontitis, or gum disease, live in the subgingival pocket between the tooth and gum surfaces, where they are in contact with a diverse microbial community, the human immune system, and a unique nutritional environment (4). The ability to directly sample communities from the human subgingival environment has provided a real-time snapshot of microbial gene expression during periodontitis, including establishing the importance of virulence factor expression and butyrate synthesis in periodontitis at the community level (5, 6). However, the majority of these studies have not had the depth and breadth to examine the gene expression of individual taxa within the human oral cavity (7).

Here, we performed a meta-analysis of 93 human oral metatranscriptomes, focusing on the gene expression patterns of the oral pathogen *Porphyromonas gingivalis* (8). *P. gingivalis* is an obligate anaerobe and is asaccharolytic, using amino acids as its primary carbon source. This microbe is often associated with chronic periodontitis, and it has been characterized as a keystone pathogen because of its ability to alter the oral immune environment, leading to dysbiosis of the microbial community as a whole (9). We discovered that *P. gingivalis* highly expressed a number of virulence factors, including the Arg- and Lys-gingipains, and genes related to growth and metabolism during periodontal disease. However, a comparison to 122 transcriptomes from experimental model systems revealed that the *P. gingivalis* periodontitis transcriptome was not distinct from growth in some common laboratory conditions, specifically logarithmic growth in rich media. Finally, we showed that the global conservation of *P. gingivalis* gene expression between periodontitis samples and certain laboratory environments is related to low variance in gene expression across environments for *P. gingivalis* in contrast to other pathogens.

## Results

### Validation of Mapping Approach and *P. gingivalis* Pangenome.

**Fig. 1.**
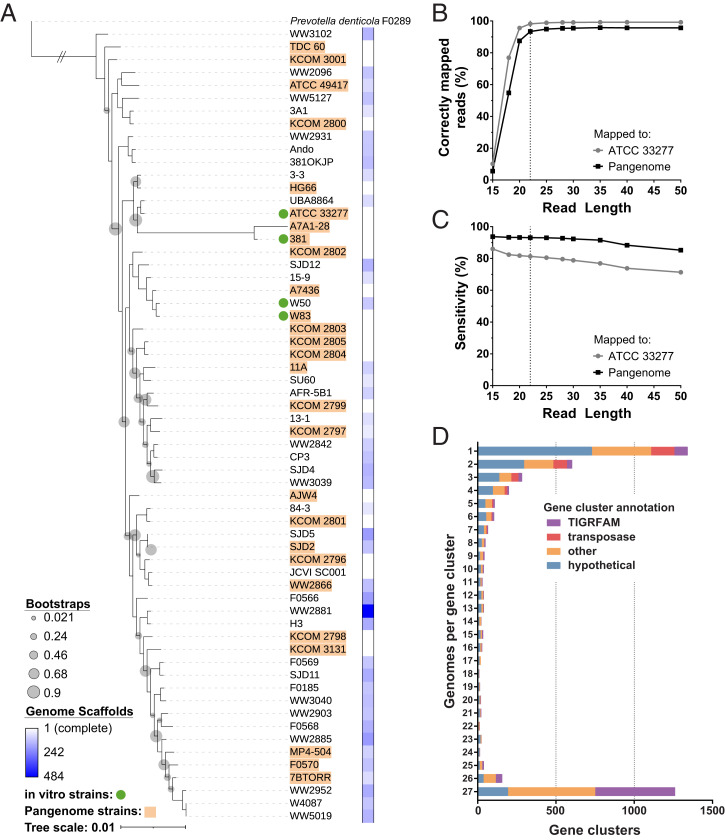
Pangenome analysis for metatranscripome analyses. (*A*) Maxiumum Likelihood phylogeny for 62 *P. gigivalis* strains and the outgroup *Prevotella denticola* F0289 built using an alignment of 49 core genes in FastTree2. The 27 strains included in the pangenome are highlighted in orange. Bootstraps less than 0.9 are shown with gray bubbles. The completeness of the genome is indicated by the number of genome scaffolds ranging from white (complete genome) to blue (484 genome scaffolds). (*B*) The percentage of mapped reads that originated from *P. gingivalis* at each read length in a mock metatranscriptome analysis. A total of 10 mock metatranscriptomes were built with read lengths varying from 15 to 50 bp using 658 genomes in the Human Oral Microbiome Database. These mock metatranscriptomes were each mapped to a single *P. gingivalis* strain (ATCC 33277) and to the pangenome of 27 strains. (*C*) The percentage of *P. gingivalis* reads in the mock metatranscriptome that aligned to the genome or pangenome at each read length. Based on the analysis here and in *B*, 22 bp was chosen as the minimum read length for an analysis of the biological samples as indicated by the dashed lines. (*D*) Distribution and annotation of the 4,643 ortholog clusters in our pangenome of 27 *P. gingivalis* strains. Colors indicate if the ortholog cluster had a TIGRFAM annotation and, if not, if it was annotated as a transposase, another function (other), or hypothetical.

The goal of this work was to analyze the gene expression of *P. gingivalis* in the human oral cavity. A challenge in this and similar analyses is that metatranscriptomes include short sequencing reads from diverse microbes. Thus, it is important to ensure sequencing reads are assigned to the correct microbe. In our case, human oral subgingival metatranscriptomes often contain reads from over 100 species and may contain multiple strains of *P. gingivalis*. Our objective was to identify *P. gingivalis*–derived reads from across the strain diversity of this species while minimizing the inclusion of non–*P. gingivalis*–derived reads. Correct mapping depends on a number of factors, including sequence read length and the identity of the reference sequence. To test these two factors, we built 10 mock oral metatranscriptomes, each containing 658 bacterial genomes from the Human Oral Microbiome Database and each with a different read length ranging from 15 to 50 base pairs (bp). Then, we mapped the mock metatranscriptomes to the *P. gingivalis* American Type Culture Collection (ATCC) 33277 genome and to a pangenome of *P. gingivalis* strains. This pangenome was constructed to represent the taxonomic diversity of the species using 27 high-quality genomes (Fig. 1*A* and Dataset S1*A*). For the mock metatranscriptome with 15-bp reads, 25% of all the reads mapped to *P. gingivalis* ATCC 33277, but only 10% of mapped reads originated from *P. gingivalis*, indicating high levels of mapping from other members of the in silico community (Fig. 1*B*). However, as the read length in the metatranscriptome increased, the percentage of mapped reads originating from *P. gingivalis* increased, plateauing at 99% with 25-bp reads and longer. When mapped to the pangenome, the pattern was similar, with at least 95% of mapped reads originating from *P. gingivalis* at 25-bp read length and longer. Based on this analysis, we chose 22 bp as the minimum read length for our biological samples to balance the specificity of mapping with the short reads in many of the biological samples (Fig. 1*B*, Dataset S2*A*, and *SI Appendix*, Table S1). Although this approach may detect some reads from other microbes in the community, it is highly selective for *P. gingivalis* reads. Furthermore, the average read length of the biological samples analyzed below was 61 bp, and the 50-bp mock metatranscriptome analysis shows that these longer reads provide additional stringency (Fig. 1*B* and *SI Appendix*, Table S1). Additionally, we asked how many of the total *P. gingivalis* reads in the mock metatranscriptome were identified via mapping. When we aligned the 22-bp mock metatranscriptome to the single *P. gingivalis* strain, 81% of the total *P. gingivalis* reads mapped, but when we aligned to the pangenome, 94% of the total *P. gingivalis* reads mapped (Fig. 1*C* and *SI Appendix*, Table S1). Therefore, we identified more of the *P. gingivalis* reads when mapping to the pangenome, likely because of the additional accessory genes in the pangenome, and we chose to map our biological samples to the *P. gingivalis* pangenome.

After deciding on the minimum read length of 22 bp and to map to the pangenome, we next defined orthologous genes across all 27 genomes in the pangenome. This step was important to allow for gene-based analyses of the biological samples. We identified 4,643 clusters of genes in our *P. gingivalis* pangenome (Fig. 1*D* and Dataset S1). Note that the 27 *P. gingivalis* genomes each have 1,929 protein coding genes on average. A total of 1,261 clusters included genes from all 27 genomes, and 1,342 clusters only included a single gene (Fig. 1*D*). Only 16 clusters contained multiple genes from the same genome. A total of 85% of the clusters with genes from all 27 genomes have a functional annotation, while 54% of the clusters containing only one gene are annotated as hypothetical, indicating that many of the known functions are represented by the shared orthologs. Collectively, these orthologs and the mock metatranscriptome benchmarking provide a rigorous and validated approach to analyze the *P. gingivalis* transcriptional profile in complex metatranscriptomes.

### *P. gingivalis* Transcripts Are Enriched in Metatranscriptomes from One-Third of Periodontally Diseased Patients.

**Fig. 2.**
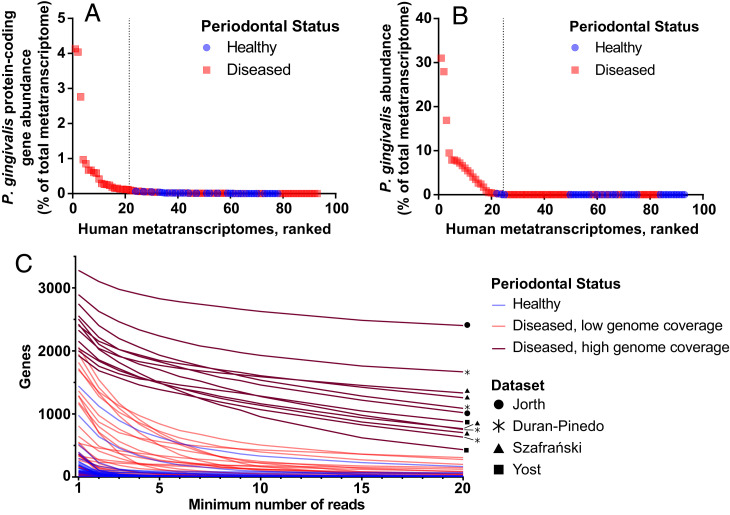
*P. gingivalis* relative transcriptional abundance across 32 periodontally healthy and 61 periodontally diseased patient samples. (*A*) *P. gingivalis* relative abundance based on the proportion of metatranscriptome reads that mapped to the pangenome of *P. gingivalis* protein-coding genes. Samples are ranked based on abundance, and the dashed line separates samples with *P. gingivalis* relative abundance less than 0.1%. (*B*) *P. gingivalis* relative abundance as determined by MetaPhlAn 3. Samples are ranked based on *P. gingivalis* abundance, and the dashed line separates samples with *P. gingivalis* relative abundance less than 0.1%. (*C*) The read coverage of the *P. gingivalis* pangenome genes for each metatranscriptome. Each line represents the relationship between gene coverage and read depth for a single metatranscriptome. The 12 periodontitis samples with high genome coverage depth and breadth that were chosen for detailed analyses are colored in dark red and labeled with the dataset name (Table 1).

We analyzed 93 previously published metatranscriptomes from six studies for *P. gingivalis* transcripts (Table 1 and Dataset S2*A*) (10–15) using the framework determined using the mock metatranscriptomes above and shown in *SI Appendix*, Fig. S1. These metatranscriptomes included 61 periodontally diseased samples from 38 individuals, including patients with gingivitis, aggressive periodontitis, and chronic periodontitis. In addition, the metatranscriptomes included 32 periodontally healthy samples, each from a separate patient. Most of the samples were collected directly from the human subgingival pocket, but 10 diseased and 10 healthy samples were from saliva. For each metatranscriptome, we mapped all reads longer than 22 bp to the pangenome of 27 *P. gingivalis* strains. Reads mapping to *P. gingivalis* protein-coding genes constituted at least 0.1% of the total metatranscriptome in 21 of 61 diseased samples (34%) but not in any of the 32 healthy samples (Fig. 2*A* and Dataset S2*A*). Reads mapping to *P. gingivalis* tended to be more abundant in diseased samples (Mann–Whitney *U* test, *P* = 0.07). Similarly, MetaPhlAn identified that *P. gingivalis* constituted at least 0.1% of the community in 22 of 61 periodontally diseased samples but only 1 of 22 periodontally healthy samples and that *P. gingivalis* was significantly enriched in the diseased samples (Mann–Whitney *U* test, *P* = 0.002; Fig. 2*B* and Dataset S2). In addition, *P. gingivalis* was the dominant *Porphyromonas* species across diseased samples (Dataset S2*B*). It is important to note that these findings represent the transcriptional abundance of *P. gingivalis*, and transcript levels do not always correlate with DNA levels in the oral cavity (12). By analyzing the number of genes with mapped reads in each sample, we narrowed our study to 12 human diseased samples with high coverage of the *P. gingivalis* pangenome (Fig. 2*C*). These 12 samples are from four different publications and from both chronic and aggressive periodontitis patients. In addition, these samples are each dominated by a single strain of *P. gingivalis*, but across these samples, there is diversity in the dominant strain (*SI Appendix*, Fig. S2).

**Table 1.** Human oral metatranscriptomic datasets

Reference	Diseased samples	Healthy samples
Belstrøm et al., *NPJ Biofilms and Microbiomes*, 2017. (saliva) (10)	10 (chronic periodontitis)	10
Duran-Pinedo et al., *ISME J*, 2014 (11).	7 (chronic periodontitis)	6
Jorth et al., *mBio*, 2014 (12).	3 (aggressive periodontitis)	3
Nowicki et al., *mBio*, 2018 (13).	3 (gingivitis)	3
Szafrański et al., *NPJ Biofilms and Microbiomes*, 2015 (14).	6 (chronic periodontitis)	10
Yost et al., *Genome Medicine*, 2015 (15).	16 (chronic periodontitis, stable site); 16 (chronic periodontitis, progressing site)	0

We also compared the clinical parameter, pocket depth, to *P. gingivalis* relative abundance (Dataset S2*A*). Pocket depth measures the separation between the gum and tooth and is one assessment of disease severity; a pocket depth of 1 to 3 mm often indicates periodontal health or gingivitis, while a pocket depth ≥ 4 mm often indicates periodontitis (16). All samples with high coverage of the *P. gingivalis* genome that were chosen for downstream analysis had a pocket depth of at least 5 mm (*SI Appendix*, Fig. S3). Together, this meta-analysis of 93 human metatranscriptomes shows that *P. gingivalis* transcripts are detected rarely in healthy samples and are more abundant in periodontally diseased samples.

### Housekeeping and Virulence Functions Are Highly Expressed in Periodontitis.

**Fig. 3.**
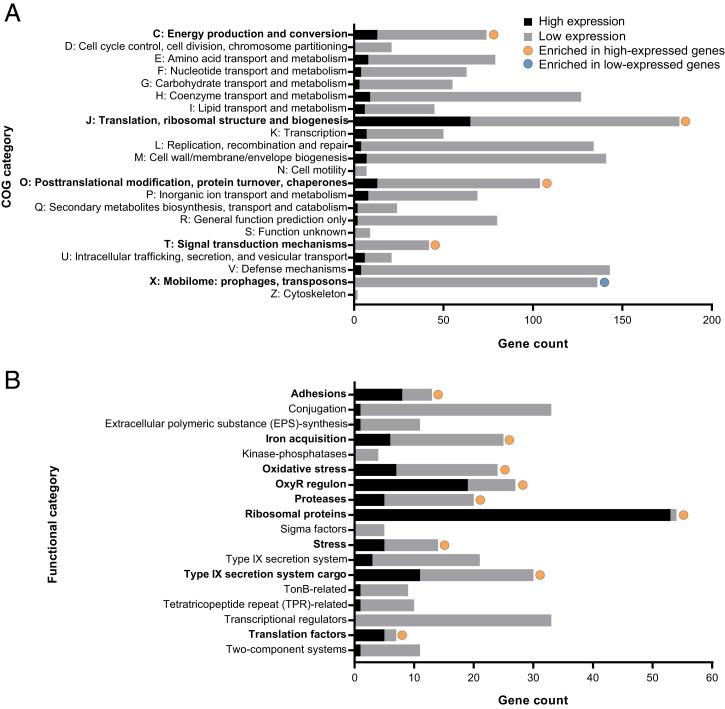
Functional enrichment of highly expressed *P. gingivalis* genes during periodontitis. Enrichment of COG categories (*A*) and hand-curated functional categories (*B*) was determined using a two-sided Fisher’s exact test, and significance is shown for *P_adj_* < 0.05. High expression genes are the 258 genes with the highest mean expression across the 12 periodontitis samples (*SI Appendix*, Fig. S4), while the low expression genes are the remaining genes.

To probe the physiology of *P. gingivalis* during periodontitis, we first analyzed the function of the 258 most highly expressed *P. gingivalis* genes on average across the 12 periodontitis samples. This gene set was chosen using an inflection point analysis of the ranked average transcripts per kilobase million (TPM) gene expression counts (*SI Appendix*, Fig. S4 and Dataset S3). These genes were enriched for clusters of orthologous groups of protein (COG) categories C (energy production and conversion), J (translation, ribosomal structure, and biogenesis), O (posttranslational modification, protein turnover, and chaperones), and U (intracellular trafficking, secretion, and vesicular transport) (Fig. 3*A*). In addition, COG category X (Mobilome: prophages and transposons) was underrepresented in the highly expressed genes, although one putative IS982 family transposase not classified by COG was among the highly expressed genes. Thus, much of the highly expressed genes were involved in key cellular functions such as translation, protein secretion, and ATPase activities.

This finding was further supported using hand-annotated functional categories of genes known to be important for growth, pathogenicity, and colonization (Fig. 3*B* and Dataset S3) with the caveat that, in this classification scheme, many genes are assigned to multiple functional categories. A total of 53 of 54 ribosomal proteins (98%) were among the top 258 most highly expressed genes, as were 19 of 27 genes (70%) annotated as part of the oxidative stress regulon, including the oxidative stress-responsive regulator OxyR. Functions related to pathogenesis were also significantly enriched. A total of 11 genes annotated as fimbrial proteins were among the 258 most highly expressed genes, including both putative major and minor fimbrial subunits, and genes encoding proteins involved in iron acquisition were significantly enriched in the highly expressed gene set. In addition, the putative Arg- and Lys-gingipains were among the highly expressed genes (*rgpA*, *rgpB*, and *kgp*). Finally, these proteases and the citrullinating peptidylarginine deiminase (*ppad*) were among the 11 highly expressed genes classified as encoding Type IX Secretion System (T9SS) cargo proteins (11 of 30, 37%), and a gene annotated as a Type VI Secretion system needle protein Hcp was also highly expressed. This analysis shows that *P. gingivalis* is actively growing and dividing in the human oral cavity, and during periodontitis, *P. gingivalis* expresses genes that contribute to pathogenesis such as those encoding adhesins, iron acquisition proteins, proteases, and secretion system–related proteins.

### The *P. gingivalis* In Situ Transcriptional Profile Clusters with Transcriptomes from Logarithmic Growth.

An alternative approach to understanding the physiology of *P. gingivalis* in the human oral cavity is through comparison to in vitro growth conditions that have known physiologies. Thus, we compared *P. gingivalis* gene expression in the 12 periodontitis samples to 122 transcriptomes of wild-type *P. gingivalis* collected during growth in experimental model systems (Table 2 and Dataset S2*C*). A total of 117 of these transcriptomes were from in vitro culture from 12 publications, mostly from growth in rich media under laboratory conditions (17–29). Of these transcriptomes, 85 were from monoculture and 32 from pairwise coculture with either *Streptococcus gordonii*, *Acinetobacter baumannii*, or *Candida albicans*. These in vitro datasets include data from four *P. gingivalis* strains (Table 2 and Fig. 1). The remaining five transcriptomes are from a murine abscess model of infection using *P. gingivalis* ATCC 33277 and were generated for this study. To ensure that any differences in gene expression were not due to differences in gene content between the strains in the experimental model systems or the human specimens, we identified a core gene set for all comparative analyses. This core set consisted of 1,500 genes that met at least one of two criteria: 1) present in at least 26 of the 27 genomes in the pangenome and/or 2) had aligned sequencing reads in all 12 human metatranscriptomes with high *P. gingivalis* coverage and all 122 experimental model system transcriptomes (Dataset S1).

**Table 2.** *P. gingivalis* transcriptomic datasets from experimental model systems

Reference	*P. gingivalis* strain(s)	Growth conditions*	Transcriptomes
Belvin et al., *Infect Immun*, 2019 (17).	W83	Midlogarithmic phase, mycoplasma media ± nitrite	4 per condition (8 total)
Cheng et al., *Metallomics,* 2019 (18).	ATCC 33277	Logarithmic phase, THK ± ranitidine bismuth citrate added at midlogarthmic phase and then sampled at 30, 60, 180, and 360 min	4 per condition, 1 per time point (8 total)
Coats et al., *Infect Immun*, 2019 (19).	ATCC 33277 and 381	Stationary phase, TYHK	3 per strain (6 total)
Dou et al., *Mol Oral Microbiol*, 2018 (20).	W83	Late-logarithmic phase, BYHK with hydrogen peroxide	3 total
Hendrickson et al., *Front Microbiol*, 2017 (21).	ATCC 33277	Pelleted cells, held anaerobically in phosphate-buffered saline for 0, 5, 30, 60, 120, 240, and 360 min ± *S. gordonii* DL1, ± DMSO, or ± 4-aminobenzoate	2 to 3 per condition and time point (36 total)
Hovik et al., *J Bacteriol*, 2012 (22).	W83	Hard agar, blood agar plates + HK; midlogarithmic phase, THK; midlogarithmic phase, chemically defined minimal liquid media	1 per condition (3 total)
Jain et al., *J Bacteriol*, 2019 (23).	ATCC 33277	Stationary phase, TYHK	3 total
Kin et al., *J Oral Microbiol*, 2020 (24).	W50	Logarithmic phase, Oral Bacterial Growth Medium ± *Treponema denticola* spent media	3 per condition (6 total)
Miller et al., *Mol Oral Microbiol*, 2018 (25).	ATCC 33277	Pelleted cells, held aerobically in phosphate-buffered saline for 180 min ± *A. baumannii* AB0057	4 monoculture, 8 coculture (12 total)
Moradali et al., *ISME J*, 2019 (26).	W83 and 381	Hard and soft agar, 0.3 and 1.5% blood agar plates + THK	3 per condition per strain (12 total)
Moye et al., *Appl Environ Microbiol*, 2019 (27).	W83	Midlogarithmic phase, THK ± 0.1% galactose	3 per condition (6 total)
Shen et al., *Mol Oral Microbiol*, 2020 (28).	ATCC 33277	Logarithmic phase, TYHK	5 total
Sztukowska et al., *mBio*, 2018 (29).	ATCC 33277	Logarithmic phase, CaGHK ± *C. albicans* hyphae or *C. albicans* spent media	3 per condition (9 total)
This study.	ATCC 33277	Murine inner thigh abscess model	5 total

*Abbreviations: T = tryptic soy broth; B = brain–heart infusion; H = hemin; K = vitamin K; Y = yeast extract; DMSO = dimethyl sulfoxide; CaG = yeast nitrogen base, 10 mM NaH_2_PO_4_ buffer (pH 7.0), 0.05% Bacto tryptone, and 0.4% glucose.

**Fig. 4.**
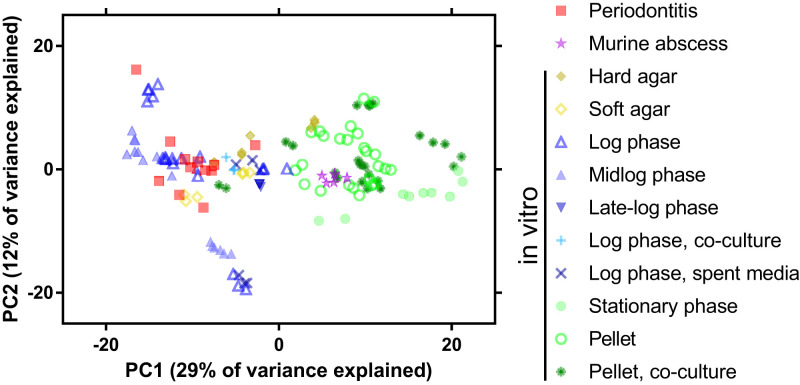
PCA of the *P. gingivalis* transcriptome in human periodontitis samples and experimental model systems. PCA was constructed using rlog-normalized read counts. Sample shapes and colors indicate the growth condition. Logarithmic growth is indicated when the methods were unclear on whether the culture was early-, mid-, or late-logarithmic phase. Pellet indicates cells that were pelleted then held statically. See *SI Appendix*, Fig. S5 for sample annotation by publication and strain and for the scree plot.

A principal component analysis (PCA) of the regularized log (rlog)-normalized gene expression across these samples showed that the *P. gingivalis* periodontitis transcriptomes do not cluster separately from the in vitro transcriptomes (Fig. 4 and *SI Appendix*, Fig. S5). This finding suggests that at a global level, *P. gingivalis* messenger RNA levels are not distinct between in vitro culture and the human oral cavity. However, the samples did separate across the first principal component (PC1). Specifically, the periodontitis transcriptomes clustered with in vitro transcriptomes collected during logarithmic phase growth or on agar plates and distinct from transcriptomes collected during stationary phase growth, pelleted cells, or the murine abscess (Fig. 4). Of note, both the soft and hard agar samples were from actively growing cultures collected 24 to 30 h after inoculation, before significant colony biofilm formation (26). These findings are supported by a heatmap of Euclidian distances between the read counts of the samples in which the periodontitis samples cluster with midlogarithmic phase and soft agar transcriptomes and separately from cultures grown to late-logarithmic or stationary phase, collected from cell pellets, or harvested from murine abscesses (*SI Appendix*, Fig. S6). In the Euclidian distance and PCA, the experimental model systems also cluster strongly by the individual RNA sequencing (RNA-seq) studies from which the data were obtained (*SI Appendix*, Figs. S5*A* and S6). However, the model systems do not cluster by the *P. gingivalis* strain used in the experiments (*SI Appendix*, Figs. S5*B* and S6).

We further identified the genes that had strong correlations with PC1 in the PCA, as this axis separated the samples by growth condition. Putative stress-related genes, including those annotated as RpoE, universal stress protein, and DNA starvation/stationary phase protection protein were positively correlated with PC1, as were transposases and a number of genes encoding proteins with T9SS sorting domains, indicating that these functions had higher expression in stationary phase grown cells, pelleted cells, and within murine abscesses than in the periodontitis metatranscriptomes or during logarithmic growth. In contrast, genes encoding for the heme import system (HmuRY), tetrahydrofolate metabolism, histidine degradation, and transcription- and translation-related proteins were negatively correlated with PC1, indicating that these functions had higher expression in periodontitis and logarithmic growth datasets than in the stationary phase, pelleted, and murine abscess datasets. Thus, while *P. gingivalis* does not have a distinct periodontitis transcriptome, cells in situ are more transcriptionally similar to those in actively growing cultures than those in nutrient-limited, nongrowing cultures or in the murine abscess.

### Growth in Midlogarithmic Phase and on Agar Plates Are Highly Transcriptionally Accurate Infection Models.

To quantify the extent to which *P. gingivalis* gene expression in each model mimics that in the human oral cavity, we used an accuracy score (AS) framework recently developed in our laboratory (30). This quantitative framework determines the fraction of genes in an experimental model whose normalized expression falls within a specified number of SDs of the mean of the expression in situ (periodontitis in this case), providing an easily interpretable gauge of model performance. As in our previous studies, we have chosen to focus on genes whose expression falls within two SDs (termed AS_2_) of the mean in the in situ metatranscriptomes, as two SDs encompass the expression range of ∼95% of the in situ samples for each gene. For example, if a model has an AS_2_ of 90%, then expression of 90% of a microbe’s genes fall within two SDs of the means of the genes in the in situ metatranscriptomes. While the AS score can be calculated using other SD ranges, such as an AS_1_ or AS_1.5_, the false-negative rate increases with more stringent criteria; for example, an AS_1.5_ has a false-negative rate of ∼13%, while the false-negative rate for AS_2_ is ∼5% (30). This AS metric is valuable in that it uses *P. gingivalis* periodontitis gene expression as a benchmark, and it determines accuracy of model systems using all of the core genes.

**Fig. 5.**
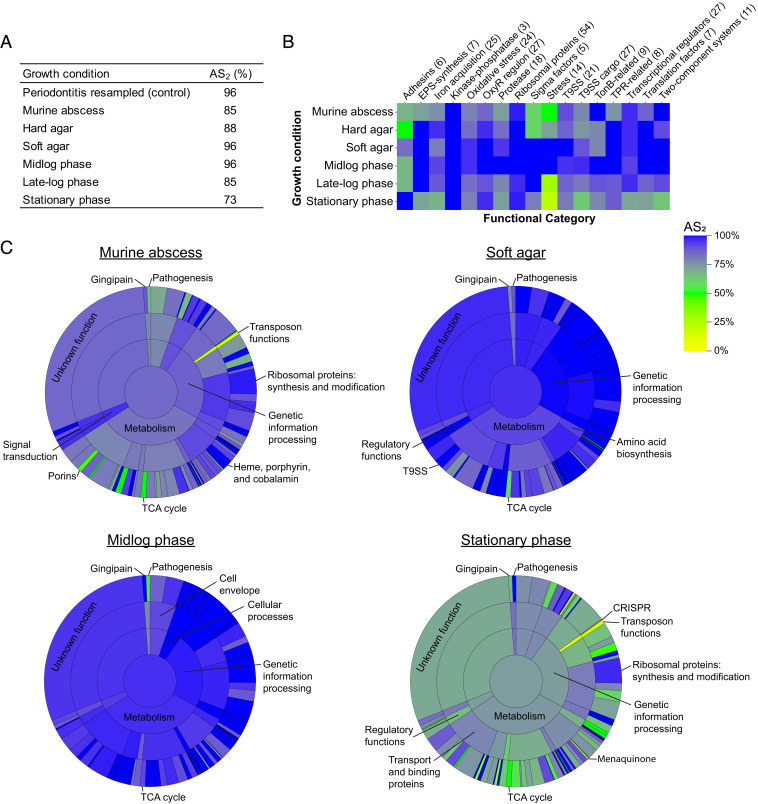
Accuracy of *P. gingivalis* gene expression in experimental model systems relative to periodontally diseased patient samples. (*A*) Accuracy of relevant growth conditions compared to gene expression during periodontitis based on the percentage of genes that fall within two SDs of the mean gene expression during periodontitis (AS_2_). Periodontitis resampled is a control to measure the upper AS_2_ benchmark, in which the AS_2_ was calculated for 500 randomly resampled pairs of periodontitis transcriptomes. (*B*) AS_2_ for hand-curated functional categories across growth conditions. Heatmap depicts the AS_2_ for within each functional category. The number of genes in each functional category is indicated in parentheses. Abbreviations: EPS = extracellular polymeric substance; T9SS = type IX secretion system; TPR = tetratricopeptide repeat. (*C*) AS_2_ for hierarchical TIGRFAM functional categories across growth conditions. “Sunburst” plots depict the AS_2_ at each hierarchical TIGRFAM level for the indicated experimental model systems. The center circle represents the AS_2_ for all core genes, the innermost ring represents the AS_2_ for TIGRFAM meta roles, the middle ring represents the AS_2_ for TIGRFAM main roles, and the outermost ring represents the AS_2_ for sub roles. The area of each section corresponds to the number of genes in that functional category. See *SI Appendix*, Fig. S7 for plots for hard agar and late-logarithmic phase growth.

Before assessing the accuracy of model systems, we performed a control experiment to assess the accuracy of subsampled *P. gingivalis* periodontitis transcriptomes with the rationale that these analyses will provide an upper AS_2_ benchmark for the experimental models. To accomplish this, we calculated the AS_2_ for *P. gingivalis* gene expression in the periodontitis metatranscriptomes by randomly choosing two *P. gingivalis* periodontitis transcriptomes as the “model” and comparing their gene expression to the remaining 10 periodontitis transcriptomes and then repeating this analysis 500 times. This analysis resulted in a mean AS_2_ value of 96%, indicating that across the periodontitis transcriptomes, 96% of the core genes fell within two SDs of the mean expression (Fig. 5*A*, periodontitis resampled). These results set the upper benchmark for model systems at 96%, as this is the accuracy of the periodontitis samples themselves.

Next, we calculated the AS_2_ for common growth models (Fig. 5*A*, and Dataset S3*D*). The AS_2_ values for liquid midlogarithmic growth and soft agar were 96%, equal to the benchmark AS_2_ of the periodontitis samples. Thus, these *P. gingivalis* in vitro growth conditions are indistinguishable from periodontitis transcriptomes using this metric. In contrast, in the murine abscess, 85% of the *P. gingivalis* genes fell within two SDs of the mean periodontitis expression, and stationary phase growth had the lowest AS_2_ of 73%. When the AS_2_ was calculated for individual replicates, the *P. gingivalis* transcriptional profile during growth in soft agar and in liquid midlogarithmic phase were significantly more accurate than that in stationary phase (Dunn’s multiple comparison test, *P_adj_* < 0.001), and growth on soft agar was significantly more accurate than in murine abscesses (Dunn’s multiple comparison test, *P_adj_* = 0.04; *SI Appendix*, Fig. S7*A*).

### Accuracy of Experimental Growth Conditions across Functional Categories.

To understand the transcriptional changes driving these differences in accuracy score, we determined the AS_2_ for individual functional categories (Fig. 5 *B* and *C*, *SI Appendix*, Fig. S7*B*, and Dataset S3*D*). These categories include the hand-curated functional classifications used in Fig. 3*B* and hierarchical TIGRFAM categories, which were hand curated to assign putative sub roles, main roles, and meta roles for all core *P. gingivalis* genes (Dataset S1) (30–32). Transcript levels of metabolic functions were highly similar between midlogarithmic phase growth and periodontitis, but metabolism is largely dysregulated in stationary phase relative to periodontitis (Fig. 5*C*). An especially inaccurate metabolic functional category was the tricarboxylic acid (TCA) cycle, which only had high accuracy during midlogarithmic growth (AS_2_ = 92%) and was the least accurate functional category during growth on soft agar (AS_2_ = 58%). Low accuracy in the TCA cycle in most growth conditions was due to lower expression in periodontitis of an oxaloacetate decarboxylase, three subunits of the fumarate reductase/succinate dehydrogenase, three subunits of the 2-oxoglutarate oxidoreductase, and an adjacent ferredoxin. In addition, the TIGRFAM meta role “genetic information processing” had decreased accuracy in stationary phase relative to other conditions, including low AS_2_ values for transposon functions (13%, overexpressed relative to periodontitis), protein stabilization–related genes (47%, predominately overexpressed relative to periodontitis), and transfer RNA aminoacylation genes (60%, both over- and underexpressed relative to periodontitis; Fig. 5*C*). This is also evident in the low accuracy of the “stress” functional category (AS_2_ = 21%), which includes overexpressed genes in stationary phase relative to periodontitis encoding for chaperones, Clp protease, universal stress protein, and DNA starvation/stationary phase protection protein (Fig. 5*B*). However, ribosomal proteins and oxidative stress genes within the OxyR regulon had high accuracy across growth conditions (Fig. 5 *B* and *C*).

Virulence-related functions also varied in their accuracy scores. The T9SS AS_2_ values ranged from 77% in stationary phase transcriptomes to 100% for soft agar transcriptomes (Fig. 5 *B* and *C*). In addition, the accuracy scores varied for genes encoding the Arg- and Lys-gingipains and gingipain-associated proteins; the gingipain functional category had an AS_2_ of 100% in midlogarithmic phase transcriptomes but 67% in stationary phase. Finally, adhesins (Fig. 5*B*) and pathogenesis (Fig. 5*C*) had low accuracy in mid- and late-logarithmic phase and a higher accuracy in stationary phase. It is likely that strain differences in fimbriae expression influence these differences, as stationary phase transcriptomes included in the accuracy score analysis were derived using the closely related *P. gingivalis* strains ATCC 33277 and 381, while the transcriptomes from mid- and late-logarithmic growth were derived using strain W83 (Fig. 1 and Table 2), and W83 is known to have lower fimbriae expression (33). However, strain differences did not fully explain low accuracy in the adhesin and pathogenesis categories, as the murine abscess, infected with *P. gingivalis* ATCC 33277, had an AS_2_ of 67% in both adhesins and pathogenesis functional categories. Also, hard agar and soft agar each have replicates from both strain types, but differ in their AS_2_ for these functions.

We also identified four genes that were not accurately captured by any of the experimental models within two SDs. These genes encode three hypothetical proteins, including one conserved hypothetical protein thought to be involved in iron acquisition, and rubrerythrin, which is important for oxidative stress. Together, this analysis shows that across the majority of functional categories, midlogarithmic growth in liquid culture and growth on soft agar largely recapitulate the *P. gingivalis* gene expression patterns in the human oral cavity.

### *P. gingivalis* Gene Expression Has Low Variance.

**Fig. 6.**
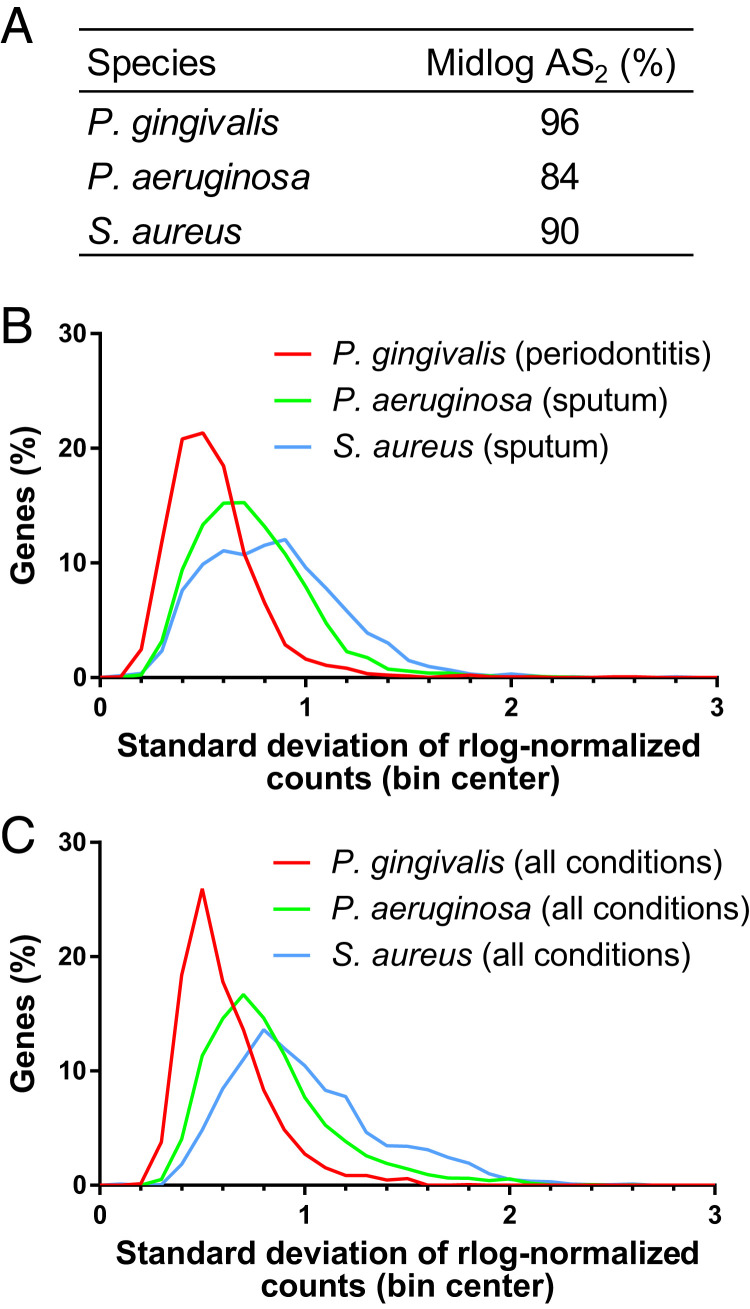
Gene expression variance of *P. gingivalis*, *P. aeruginosa*, and *S. aureus* in human patient samples and in laboratory models. (*A*) The percentage of genes that fall within two SDs of the mean in situ gene expression (AS_2_) during midlogarithmic growth. For *P. gingivalis*, 16 in vitro transcriptomes collected during midlogarithmic growth were compared to 12 periodontitis metatranscriptomes; for *P. aeruginosa*, 11 in vitro transcriptomes collected during midlogarithmic growth were compared to 20 sputum metatranscriptomes from people with cystic fibrosis; and for *S. aureus*, 14 in vitro transcriptomes collected during midlogarithmic growth were compared to 10 sputum metatranscriptomes from people with cystic fibrosis. (*B*) Histogram of the variation in gene expression across human samples. Histogram was constructed with a bin size of 0.1 using the variation in rlog-normalized bacterial gene expression levels across metatranscriptomes collected from periodontitis (*P. gingivalis*; *n* = 12) and from cystic fibrosis sputum (*P. aeruginosa* and *S. aureus*; *n* = 20 and *n* = 10, respectively). (*C*) Histogram of the variation in gene expression across diverse growth conditions. Histogram was constructed with a bin size of 0.1 using rlog-normalized bacterial gene expression levels across diverse sample types, including human metatranscriptomes, animal models, and in vitro experimental models. A total of 134 samples were analyzed for *P. gingivalis*, 92 for *P. aeruginosa*, and 34 for *S. aureus*.

Our analyses showed that for almost all functional categories, the *P. gingivalis* transcriptome was highly similar between midlogarithmic phase growth, growth on soft agar, and during periodontal disease (Figs. 4 and 5 and *SI Appendix*, Fig. S6). In contrast, similar analyses for other microbes have found distinct gene expression profiles between human infection and growth in laboratory or animal models using PCAs (1–3). These distinct profiles result in an AS_2_ of 84 and 90% for *P. aeruginosa* and *S. aureus* in midlogarithmic growth, respectively (Fig. 6*A*). In addition, an analysis of *P. aeruginosa* across diverse experimental model systems found AS_2_ values ranging from 81 to 86% for all genes (30).

Therefore, we wanted to better understand the high accuracy scores for *P. gingivalis* in certain experimental model systems. First, as the AS_2_ metric is based on the variance of the in situ gene expression, high accuracy of an experimental model system could result from high in situ gene expression variance. To ask if the high AS_2_ scores were due to high variance in *P. gingivalis* gene expression during periodontitis, we recalculated the AS_2_ scores, excluding 54 genes with a high SD during periodontitis. The resulting accuracy scores of the experimental models remained high (*SI Appendix*, Fig. S8). In addition, we found that the range of the SD of rlog-normalized read counts was smaller for *P. gingivalis* transcriptomes from periodontitis samples than for *P. aeruginosa* or *S. aureus* transcriptomes from sputum samples from people with cystic fibrosis (Fig. 6*B*). Thus, the high *P. gingivalis* accuracy scores are not due to high variance in periodontitis, and in fact, *P. gingivalis* has a smaller in situ gene expression variance than these other microbes.

Alternatively, we asked if the high accuracy scores of *P. gingivalis* were due to overall smaller deviations in gene expression for this microbe. We calculated the SD of the rlog-normalized expression of each gene for *P. gingivalis*, *P. aeruginosa*, and *S. aureus* across a wide range of growth conditions, including in situ growth, mouse models, and laboratory growth conditions (Fig. 6*C*). The SD of *P. gingivalis* gene expression had a smaller distribution than that for the other two microbes. Specifically, for *P. gingivalis*, 95% of genes across all conditions had an SD of 1 or less. In contrast, for *P. aeruginosa* and *S. aureus*, the 95th percentile was an SD of 1.5 and 1.7, respectively. Together, these findings indicate that the gene expression levels for *P. gingivalis* are more stable across growth conditions and provide insight into the high similarity of the *P. gingivalis* transcriptome between rich media in laboratory conditions and the human oral cavity.

## Discussion

Next-generation sequencing has provided a portal to understanding the behavior of microbes in their native environments. This study leverages 93 publicly available metatranscriptomes from the human oral cavity to further our understanding of the physiology of *P. gingivalis* during infection and to identify which experimental model systems best encapsulate this physiology. We discovered that *P. gingivalis* in the diseased oral cavity has highly similar gene expression to that during midlogarithmic phase in vitro growth. This finding is in contrast to previous work comparing gene expression between infection and laboratory models for diverse bacterial pathogens (1–3, 7, 30). Thus, while great effort is required to develop accurate in vitro models for other opportunistic pathogens such as *P. aeruginosa* (1, 30, 34, 35), *P. gingivalis* human gene expression is highly mimicked in a common, easily accessible laboratory model. These data also provide strong support for a need to continuously and quantitatively evaluate current model systems using sceintific data, rather than altering current models or developing new models based on intuition. We anticipate that for many bacteria, particularly niche specialists such as *P. gingivalis*, simple in vitro growth conditions may be highly useful, biologically relevant model systems.

Our discovery that *P. gingivalis* gene expression during periodontitis is similar to that in logarithmic in vitro cultures offers important context for understanding the biology of this pathogen. First, these results provide biological relevance for the thousands of in vitro and in vivo experimental results published on this bacterium. The fact that a midlogarithmic test tube model highly recapitulates gene expression in situ significantly advances our understanding of this bacterium during periodontal infection. Logarithmic growth in vitro is generally the most common system used to study *P. gingivalis* and most other bacteria, and there are decades of data on *P. gingivalis* physiology during logarithmic phase growth. Furthermore, laboratory growth in rich media offers an accessible, inexpensive experimental model for testing future hypotheses. Second, while one might expect the oral cavity to be a hostile environment because of competition for resources with other microbes and interactions with the host immune system, our findings indicate that in the periodontitis samples analyzed here, *P. gingivalis* is growing in a nutrient-rich, relatively stress-free environment. Although the transcriptional profile of *P. gingivalis* was comparably stable during periodontitis in this sample set (Figs. 4 and 6), we cannot rule out that other experimental models best capture *P. gingivalis* growth under certain conditions, for instance, at very low abundance in the oral cavity or at alternative disease sites such as the human brain in connection to Alzheimer's disease (36). Thus, this study provides a framework for future analyses of how the transcriptome of *P. gingivalis* changes relative to its abundance, across disease sites, over longitudinal studies of disease and treatment, or in the presence of different coinfecting microbes. Also, this study focused on the transcription of *P. gingivalis* during disease at the population level, primarily using core genes, and it would be interesting in the future to consider the role of strain-level differences and population-level heterogeneity as well as the accuracy of accessory genes.

In addition, we have specifically identified experimental model systems that best capture the few genes not well mimicked in logarithmic phase in vitro cultures (Fig. 5 and Dataset S3). For example, the murine abscess model was not highly accurate overall, including increased expression of stress-related genes, increased nutrient limitation, and decreased fimbria expression in the abscesses relative to periodontitis (Fig. 5 *B* and *C*); however, there are 49 genes captured by this model that were not accurately mimicked by midlogarithmic phase in vitro cultures (Dataset S3). These genes include a putative hemolysin virulence factor as well as several metabolic genes. Thus, if one is interested in this small subset of genes, the mouse would be a preferred model over midlogarithmic phase in vitro culture. Our accuracy score framework also presents future opportunities to characterize the utility of other animal models (37). Moreover, while gene expression is a major determinant of physiology, certain biological questions may additionally require the accurate capture of factors such as gene essentiality, bacterial fitness, or host–microbe interactions. For example, studying disease outcomes requires the use of animal models. Furthermore, a similar accuracy score approach could be used to compare host gene expression between human infection and animal models of periodontitis.

In our framework, the accuracy of experimental models depends both on the variance of gene expression in situ and the differences in gene expression between in situ and laboratory growth. A model could be considered accurate because its gene expression is highly similar to that in situ or because the in situ gene expression is so highly variable that it encapsulates large deviations in model gene expression. We found that *P. gingivalis* accuracy is likely due to the former, as it has low variability in gene expression across environments, including periodontitis (Fig. 6). Despite this low variability, in vitro stationary-phase cells had a low AS_2_ (73%), indicating a specific change in gene expression at this growth phase compared to periodontitis. In contrast, the high AS_2_ for *S. aureus* during midlogarithmic growth (90%) was due to highly variable gene expression within the in situ sputum samples (Fig. 6). We hypothesize that these differences in *S. aureus* and *P. gingivalis* gene expression variability result from the fact that *S. aureus* is a generalist and *P. gingivalis* a specialist. Indeed, *S. aureus* is an opportunistic pathogen that can be found in multiple sites on the body and on multiple hosts (38). In comparison, *P. gingivalis* is specific to the oral cavities of humans and old-world primates and is predominantly an oral pathogen (Fig. 2) (39). Although *P. gingivalis* has been proposed to contribute to disease at other body sites, fitness in these alternative sites is likely not a primary driver of this microbe’s evolution. Therefore, it is not unreasonable that a bacterium that is highly niche specific has evolved stable gene expression patterns to be fit in its environment, and these stable expression patterns are manifested in other, nonnative environments. We propose that *P. gingivalis* has not evolved the transcriptomic plasticity to adapt to diverse habitats, and this smaller regulatory need is also evidenced by the small regulon in *P. gingivalis* relative to many other bacteria, even relative to genome size (40, 41).

In sum, we further a recently developed framework for studying bacterial in situ gene expression, relative to experimental model systems, including the analyses to determine minimum read length for mapping and the accuracy score metric (30). Through curating hundreds of datasets, this work reveals that *P. gingivalis* gene expression during periodontitis is highly similar to midlogarithmic in vitro cultures, providing strong evidence for the use of a simple test tube model as the gold standard model for studying *P. gingivalis* biology. These results have significant implications to bacteriology, as they provide a framework to quantitatively assess the biological relevance of the tens of thousands of in vitro experiments performed in this basic laboratory model system.

## Materials and Methods

A summary of the analysis approach is available in *SI Appendix*, Fig. S1.

### Pangenome Construction.

The phylogeny of 62 *P. gingivalis* strains (all strains in The National Center for Biotechnology Information [NCBI] excluding duplicates as of January 2021) was built in Kbase using the “Build Microbial SpeciesTree” app version 1.7.1 (42). Briefly, this analysis constructs a tree using 49 core genes defined by COG gene families. These core genes were inserted into curated multiple sequence alignments for each COG family, the alignments were concatenated, and the maximum likelihood phylogenetic tree was constructed using FastTree2 version 2.1.10 (43). The tree was annotated using Interactive Tree Of Life (iTOL) v5 (44). Strains chosen for the pangenome included the 20 strains with complete genomes and seven additional genomes to ensure coverage of the species diversity (Fig. 1 and Dataset S1). Gene clusters (orthologs) were constructed for these 27 *P. gingivalis* genomes using Roary with the flag -i 90 (45). After minimal manual curation, pangenome locus tags were assigned based on the genome fragment order in the Roary output (Dataset S1). TIGRFAM annotations for each gene were assigned using KBase, Kyoto Encyclopedia of Genes and Genomes (KEGG) annotations were assigned using both BlastKOALA and KofamKOALA, and COG terms were assigned using KEGG’s binary relationships to COG in the BRITE database (46, 47).

### Mock Metatranscriptome Analysis.

We built our mock metatranscriptome using 658 genomes downloaded from the Human Oral Microbiome Database with habitat labeled as “Oral” or “Nasal, Oral” spanning 82 genera and including 19 *P. gingivalis* genomes, 10 of which are also in our pangenome (48). Art v.2.5.8 was used to simulate metatranscriptomic reads from the coding sequences of this mock community at 10 lengths varying from 15 to 50 bp with 1× coverage (49). The reads were mapped using Bowtie2 v2.3.5 to the *P. gingivalis* ATCC 33277 genome and to concatenated genomes of the 27 strains in the pangenome (50). This workflow is available at https://github.com/glew8/Pgingivalis_Metatranscriptome_Analyses.

### Murine Abscess RNA-Seq.

The murine inner thigh abscess was performed by inoculating Balb/c mice with 1.5 × 10^9^ *P. gingivalis* ATCC 33277 cells collected in the midlogarithmic growth phase and washed with sterile, prereduced phosphate-buffered saline (PBS). Abscess material was collected 48 h postinoculation by making a small incision adjacent to the abscess and collecting the secreted material using a sterile cotton swab which was then dispersed into sterile, prereduced PBS. Each sample was briefly centrifuged at 150 × *g* for 3 min at 4 °C to pellet any eukaryotic cells present from the abscess, after which the supernatant was centrifuged at 5,000 × *g* at 4 °C for 5 min to pellet bacterial cells. After removing the supernatant, the bacterial pellet was used for total RNA extraction using the Invitrogen TRIzol Max Bacterial RNA Isolation Kit (Thermo Fisher Scientific) following the manufacturer’s protocol. Ribosomal RNA depletion, strand-specific library construction, and 150-bp paired-end RNA sequencing were performed by Novogene using the Illumina NovaSeq 6000 platform.

### Quality Analyses, Mapping, and Counting of RNA-Seq Datasets.

Using only the forward reads from each dataset if paired end, RNA-seq sequencing read quality was confirmed with FastQC v0.11.8 (51). Reads were trimmed to remove adapters from the 3′ end of the reads using Cutadapt 2.6 (AGA TCG GAA GAG CAC ACG TCT GAA CTC CAG TCA C and AAG TCG GAG GCC AAG CGG TCT TAG GAA GAC AA for Illumina- and BGISEQ-sequenced reads, respectively) (52). Trimmed reads that were at least 22 bp were mapped to the concatenated genomes of 27 *P. gingivalis* strains (Dataset S1) using Bowtie2 v2.3.5 with default parameters (50). featureCounts (subread-2.0.1) was used to assign reads to protein-coding genes with the flags -s 0 (unstranded) and -O (allowMultiOverlap) in R 4.0.2 (53, 54) so that each read was assigned to a single locus or to neighboring genes. Then, read counts were summed for each ortholog to account for strain-level differences across samples. At each step, MultiQC v1.9 was used to track analysis quality (55).

### MetaPhlAn and StrainPhlAn Analyses.

The taxonomy of the human periodontitis datasets was estimated using MetaPhlAn 3.0.6 with marker gene version mpa_v30_CHOCOPhlAn_201901, the minimum read length set to 22 bp, and viruses included (56). The dominant *P. gingivalis* strain in the metatranscriptomes, relative to the 27 reference genomes in the pangenome, was determined using StrainPhlAn 3.0 with the minimum read length set to 22 bp (56, 57). iTOL v5 was used to visualize the population structure (44).

### Gene Expression Analyses.

Gene counts were normalized using TPM by dividing the raw counts by the average length for each ortholog to determine reads per kilobase (RPK), summing the RPK for each sample and dividing by 1,000,000 to determine the scaling factor, and then dividing the RPK by the scaling factor for each gene. Highly expressed genes based on TPM were identified using R package inflection version 1.3.4 (58). Enrichment was determined using a two-sided Fisher’s exact test, and *P* values were corrected for multiple testing using the Benjamini–Hochberg method in R. rlog normalization of core genes was performed on all metatranscriptomes and transcriptomes together using DESeq2 with blind = TRUE in R (59). The PCA was built using rlog-normalized counts of the 500 core genes with highest variability across samples using the command plotPCA in DESeq2. The Euclidian distance matrix was calculated from the rlog-normalized counts of the 1,500 core genes using the R function dist, and the heatmap was produced in pheatmap version 1.0.12 (60).

### AS Analyses.

Accuracy scores were calculated and graphed using rlog-normalized read counts for the 1,500 *P. gingivalis* core genes (Dataset S1*C*) in R version 4.0.2 with the following packages: tidyverse version 1.3.0, cowplot version 1.0.0, readr version 1.3.1, dplyr version 1.0.2, tidyr version 1.1.2, tibble version 3.0.3, purrr version 0.3.4, ggsunburst version 0.3.0, zeallot version 0.1.0, ggplot2 version 3.3.2, and reshape version 0.8.8 (61–71). Scripts are modified from Cornforth et al. and are available at https://github.com/glew8/Pgingivalis_Metatranscriptome_Analyses (30). This analysis calculates the mean and SD of normalized read counts for each gene in a target environment, in this case periodontitis. Then, using these values, the analysis determines the number of SDs away from the target mean gene expression for each gene in each replicate in a model (the *z*-score). The median *z*-score across replicates is identified for each gene, and this value is outputted as the “penalty.” Finally, the AS_2_ is calculated by determining the percentage of penalties that fall between −2 and 2. For AS_2_ calculations across functional categories, the approach is the same, but only the genes within the given functional category are considered. To understand how transcriptionally variable genes during periodontitis impact the AS, the AS_2_ analysis was also performed excluding 54 genes with a SD of rlog-normalized read counts greater than 1 across the 12 periodontitis samples (Dataset S3*C*).

### *P. aeruginosa* and *S. aureus* Analysis.

Raw read counts of *P. aeruginosa* transcriptomes in experimental model systems, human sputum, and human wounds were obtained from Cornforth et al. (30). *P. aeruginosa* analyses were limited to 4,945 soft core genes that were 1) present in 277 of 291 (95%) high-quality genomes as analyzed by Roary using the same parameters as the *P. gingivalis* pangenome construction above and 2) had orthologs in *P. aeruginosa* PAO1. Raw read counts of *S. aureus* transcriptomes in experimental model systems, human sputum, and human wounds were obtained from Ibberson and Whiteley (2). *S. aureus* analyses were limited to 1,960 core genes in a 15-strain pangenome (2). Raw gene counts were rlog normalized with DESeq2, and the AS_2_ analysis was performed.

## Supplementary Material

Supplementary File

Supplementary File

Supplementary File

Supplementary File

## Data Availability

RNA-seq reads from *P. gingivalis* infection for the five murine abscesses are available in the NCBI Sequence Read Archive in BioProject PRJNA762090 at https://www.ncbi.nlm.nih.gov/bioproject/762090. The other 210 datasets used in this publication are previously published as shown in Tables 1 and 2, and Dataset S2. The orthologs and curated annotations for the pangenome are available in Dataset S1. *P. aeruginosa* and *S. aureus* datasets are available from refs. 2 and 30.
