# Supplementary material for: A quantitative framework reveals traditional laboratory growth is a highly accurate model of human oral infection
Source: Proc Natl Acad Sci U S A. 2022 Jan 6;119(2):e2116637119. doi: 10.1073/pnas.2116637119 (PMC8764681; doi:10.1073/pnas.2116637119)
Supplement: Supplementary File [file pnas.2116637119.sapp.pdf]

PNAS
www.pnas.org

Supplementary Information for
A quantitative framework reveals traditional laboratory growth is a highly accurate model of human oral infection

Gina R. Lewin, Kendall S. Stocke, Richard J. Lamont, Marvin Whiteley

Marvin Whiteley
Email: mwhiteley3@gatech.edu

This PDF file includes:

Figures S1 to S8
Table S1
Legends for Datasets S1 to S3

Other supplementary materials for this manuscript include the following:

Datasets S1 to S3

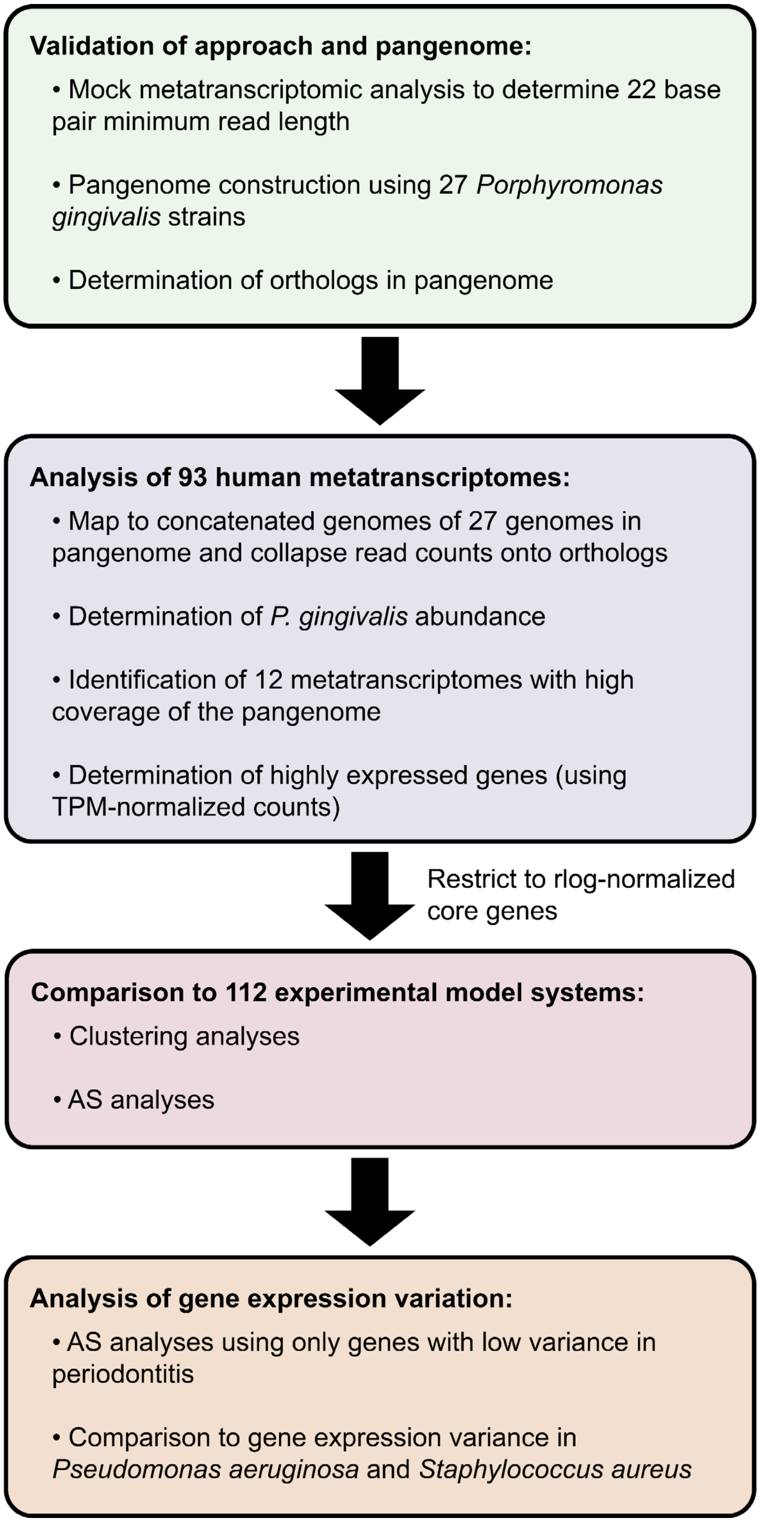

Figure S1. Flow diagram of analyses performed in this study. Abbreviations: TPM = Transcripts per Kilobase Million; rlog = regularized log; AS = Accuracy Score.

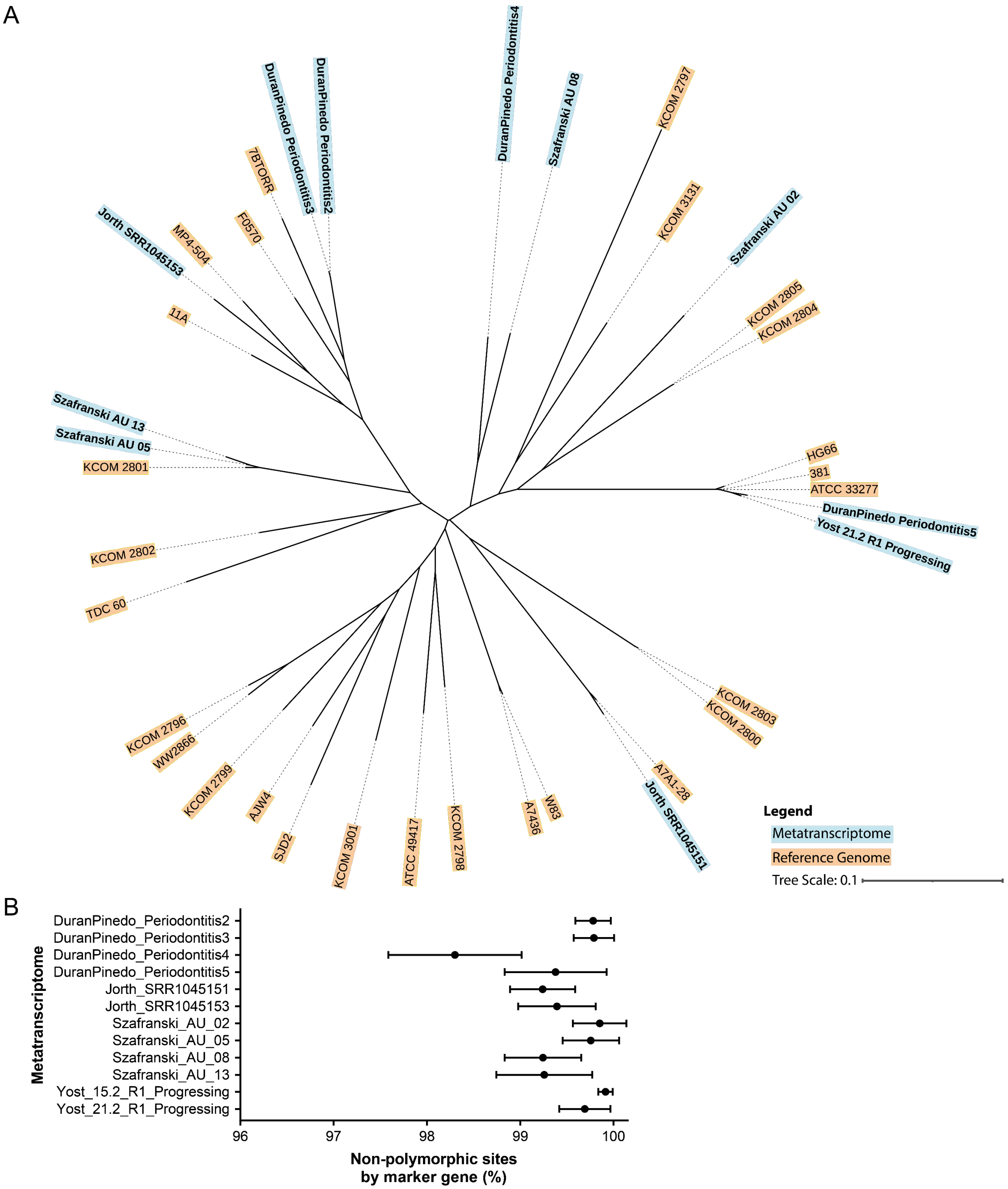

Figure S2. *Porphyromonas gingivalis* population genetic structure in periodontitis. A. The *P. gingivalis* strain population structures were determined for the focal human periodontitis samples with high coverage of the *P. gingivalis* genome, relative to the 27 *P. gingivalis* reference strains included in the pangenome using a concatenated alignment of 68 marker sequences by StrainPhlAn 3. The metatranscriptome Yost_15.2_R1_Progressing was not included in the phylogeny due to poor coverage of the marker sequences. **B.** The average and standard deviation of non-polymorphic sites across the marker genes for each metatranscriptome, as determined by StrainPhlAn 3.

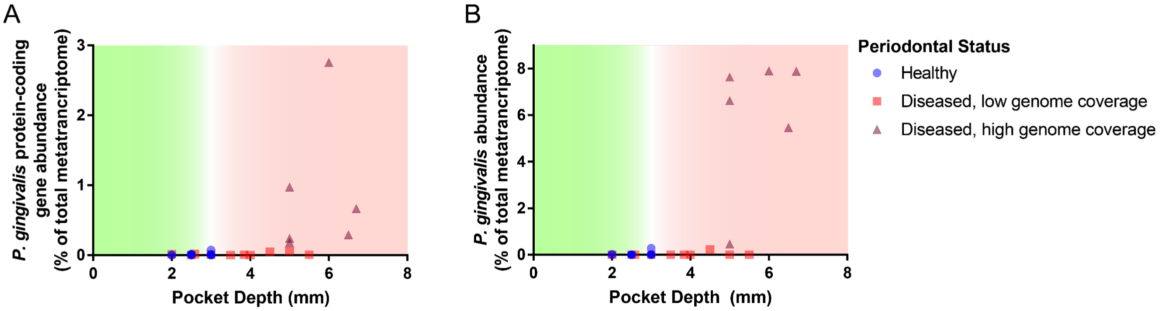

Figure S3. The relationship between measured pocket depth and *P. gingivalis* relative abundance. *P. gingivalis* relative abundance determined by **(A)** the portion metatranscriptome reads that were mapped to the pangenome of *P. gingivalis* protein-coding genes or **(B)** MetaPhlAn 3. A pocket depth of 1-3 mm (green shading) indicates healthy gums or gingivitis, while a pocket depth ≥4 (red shading) is one metric used to diagnose periodontitis.

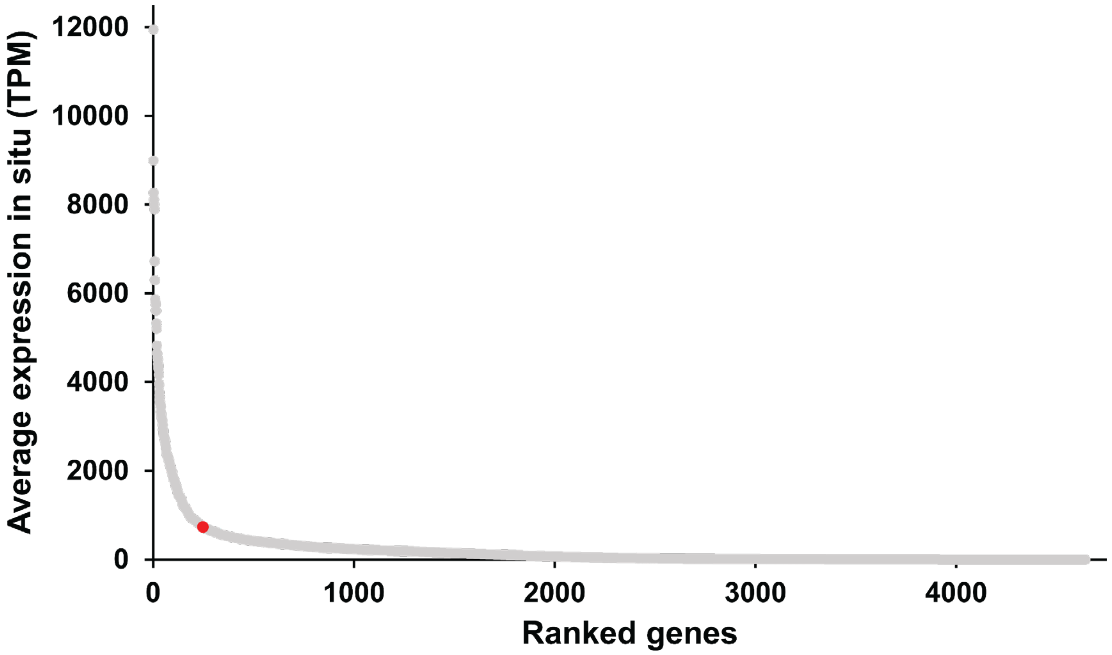

Figure S4. Ranked mean *P. gingivalis* gene expression for twelve human periodontitis samples. Read counts were normalized using Transcripts per Kilobase Million (TPM). The inflection point on the graph (258 genes) is colored red.

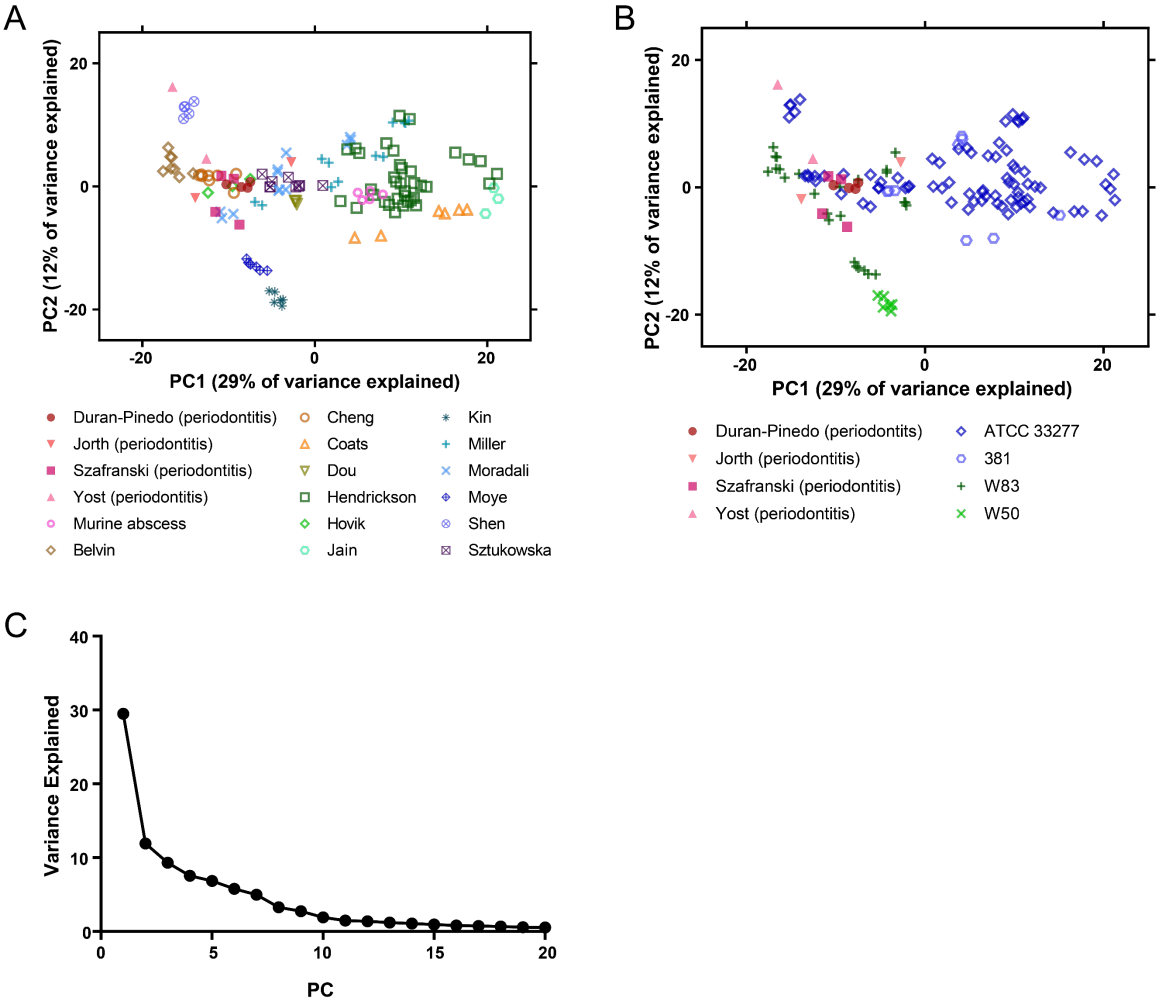

Figure S5. Principle component analysis of rlog-normalized counts of 1500 core *P. gingivalis* genes. Samples are colored based on **(A)** publication source and **(B)** strain. Strains ATCC 33277 and 381 are closely related, as are strains W83 and W50 (see Figure 1). **C.** Scree plot for the first 20 principal components (PCs).

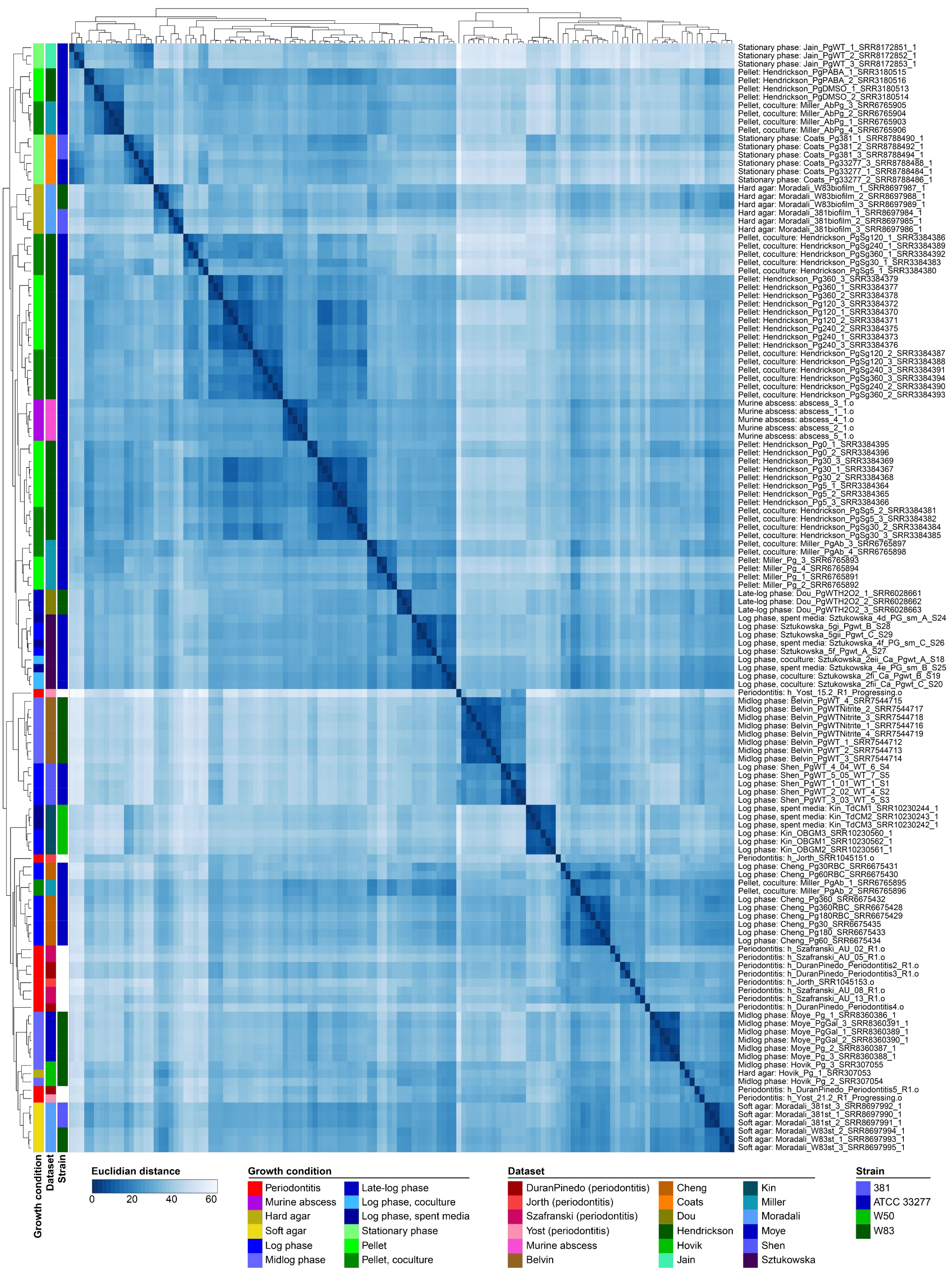

Figure S6. Heatmap of Euclidian distances of *P. gingivalis* transcriptomes using of rlog-normalized counts of 1500 core genes. Samples are ordered with hierarchical complete-linkage clustering. Growth condition, dataset (publication), and strain are shown for each sample. Strains ATCC 33277 and 381 are closely related, as are strains W83 and W50 (see Figure 1).

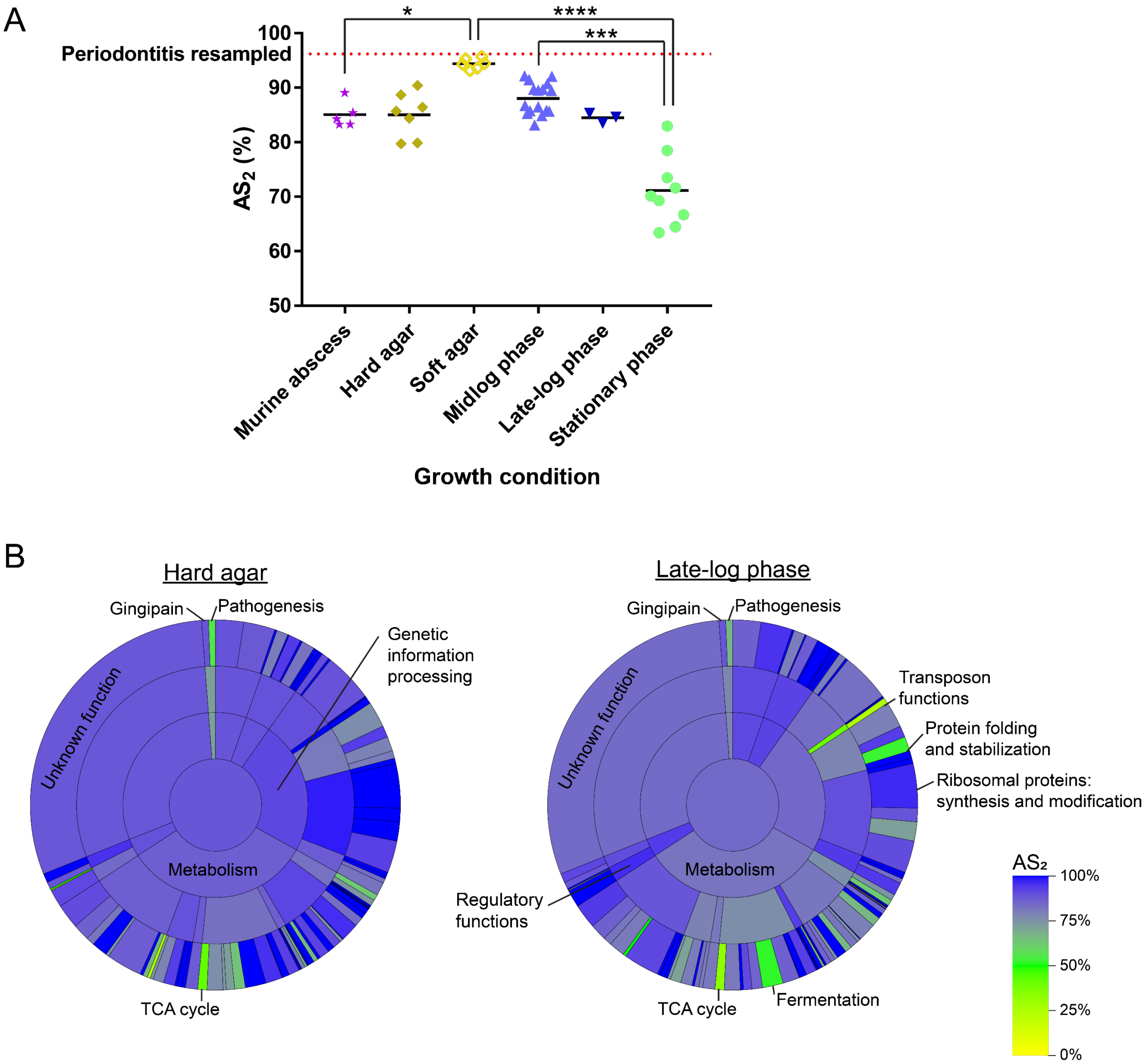

Figure S7. Accuracy of *P. gingivalis* gene expression in experimental model systems relative to periodontally diseased patient samples. A. The percentage of genes that fall within two standard deviations of the mean gene expression in periodontitis (AS_2) for individual replicates of experimental model systems. The red dashed line indicates the AS_2 for resampled periodontitis samples, a control to measure upper benchmark for the AS_2, in which the AS_2 was calculated for 500 randomly resampled pairs of periodontitis transcriptomes. *$P_{adj} < 0.05$; ***$P_{adj} < 0.0005$; ****$P_{adj} < 0.0001$, Dunn's Multiple Comparison Test. **B.** "Sunburst" plots depict the AS_2 at each hierarchical TIGRFAM level for the indicated experimental model systems. The center circle represents the AS_2 for all core genes, the innermost ring represents the AS_2 for TIGRFAM meta roles, the middle ring represents the AS_2 for TIGRFAM main roles, and the outermost ring represents the AS_2 for sub roles. The area of each section corresponds to the number of genes in that functional category. See Figure 5 for plots for other growth conditions.

A

Growth condition	AS_2 (%)
Periodontitis resampled (control)	96
Murine abscess	84
Hard agar	89
Soft agar	96
Midlog phase	96
Late-log phase	85
Stationary phase	72

B

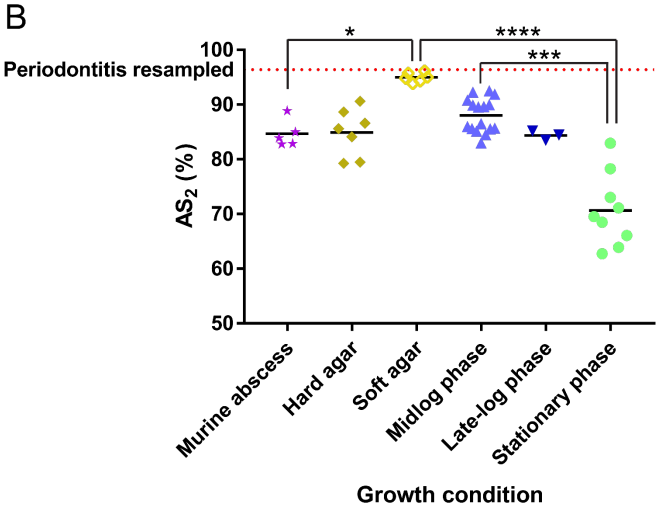

Figure S8. Accuracy of *P. gingivalis* gene expression in experimental model systems relative to periodontally diseased patient samples using low variance genes. A. The percentage of genes that fall within two standard deviations of the mean gene expression in periodontitis (AS_2) for experimental growth conditions, calculated using 1446 core genes with low variance in the periodontitis samples. Periodontitis resampled is a control to determine the upper AS_2 benchmark, in which the AS_2 was calculated for 500 randomly resampled pairs of periodontitis transcriptomes. **B.** The AS_2 for individual replicates of experimental model systems, calculated using 1446 core genes with low variance in the periodontitis samples. The red dashed line indicates the AS_2 for resampled periodontitis samples. *P_{adj} < 0.05; ***P_{adj} < 0.0005; ****P_{adj} < 0.0001, Dunn's Multiple Comparison Test.

Table S1. Metrics for mock metatranscriptome analysis mapped to *P. gingivalis* pangenome using 22 bp and 50 bp reads. Data are shown for all *Porphyromonas* species and any additional taxa contributing at least 0.1% of mapped reads.

	Metric 1*		Metric 2†	
Taxon	22 bp	50 bp	22 bp	50 bp
Porphyromonas catoniae	0.04%	0.01%	0.50%	0.13%
Porphyromonas endodontalis	0.12%	0.10%	2.61%	1.84%
Porphyromonas gingivalis	94.82%	96.84%	93.81%	85.98%
Porphyromonas pasteri	0.03%	0.01%	0.61%	0.13%
Porphyromonas sp. HMT 278	0.02%	0.01%	0.46%	0.10%
Prevotella	3.30%	2.39%	1.13%	0.73%
Alloprevotella	0.26%	0.20%	1.57%	1.09%
Tannerella	0.58%	0.35%	0.90%	0.49%
Streptococcus	0.11%	0.00%	0.02%	0.00%
Other	0.71%	0.09%	0.03%	0.00%

* reads mapped to *P. gingivalis* pangenome originating from the indicated taxon divided by the total number of mapped reads

† reads mapped to *P. gingivalis* pangenome originating from the indicated taxon divided by the total number of reads in the mock metatranscriptome for the taxon

Dataset S1 (separate file). *P. gingivalis* pangenome. A. Genomes used to build pangenome. **B.** Orthologs across pangenome. **C.** Annotation of pangenome locus tags. Includes lookup to common locus tags for strains *P. gingivalis* W83 and ATCC33277.

Dataset S2 (separate file). Datasets used in this study. A. Human oral metatranscriptomes, including detailed metadata, sequencing, mapping, counting data, and *P. gingivalis* abundance for each sample. The 12 datasets with high coverage of the *P. gingivalis* pangenome and included in downstream analyses are indicated with blue font. **B.** Community profile of the human oral metatranscriptomes at the species level, as determined by MetaPhlAn 3. Values represent percentage of the community. *P. gingivalis* abundance and the 12 datasets with high coverage of the *P. gingivalis* pangenome and included in downstream analyses are indicated with blue font. **C.** Experimental model transcriptomes (in vitro or mouse models), including metadata, sequencing, mapping, and counting data for each sample.

Dataset S3 (separate file). Sequencing count data. A. Raw counts of transcriptomes and metatranscriptomes mapped to the *P. gingivalis* pangenome. **B.** TPM-normalized counts for the 12 periodontitis datasets with high coverage of the *P. gingivalis* genome. **C.** rlog-normalized count data for all samples used in analyses. **D.** Accuracy score penalties (z-scores) across growth conditions and for individual samples.
